# A Novel Porcine Graft for Regeneration of Bone Defects

**DOI:** 10.3390/ma8052523

**Published:** 2015-05-12

**Authors:** Eisner Salamanca, Wei-Fang Lee, Chin-Yi Lin, Haw-Ming Huang, Che-Tong Lin, Sheng-Wei Feng, Wei-Jen Chang

**Affiliations:** 1 School of Dentistry, College of Oral Medicine, Taipei Medical University, 250 Wu-Hsing Street, Taipei 110, Taiwan; E-Mails: eisnergab@hotmail.com (E.S.); drjerrylin@gmail.com (C.-Y.L.); chetong@tmu.edu.tw (C.-T.L.); b8702070@tmu.edu.tw (S.-W.F.); 2 School of Dental Technology, College of Oral Medicine, Taipei Medical University, 250 Wu-Hsing Street, Taipei 110, Taiwan; E-Mail: weiwei@tmu.edu.tw; 3 Graduate Institute of Biomedical Materials & Tissue Engineering, College of Oral Medicine, Taipei Medical University, 250 Wu-Hsing Street, Taipei 110, Taiwan; E-Mail: hhm@tmu.edu.tw; 4 Dental Department of Taipei Medical University, Shuang-Ho Hospital, Taipei 110, Taiwan

**Keywords:** bone regeneration, bone graft, porcine tissue, *in vivo* test, hydroxyapatite

## Abstract

Bone regeneration procedures require alternative graft biomaterials to those for autogenous bone. Therefore, we developed a novel porcine graft using particle sizes of 250–500 μm and 500–1000 μm in rabbit calvarial bone defects and compared the graft properties with those of commercial hydroxyapatite (HA)/beta-tricalcium phosphate (β-TCP) over eight weeks. Surgery was performed in 20 adult male New Zealand white rabbits. During a standardized surgical procedure, four calvarial critical-size defects of 5 mm diameter and 3 mm depth were prepared. The defects were filled with HA/β-TCP, 250–500 μm or 500–1000 μm porcine graft, and control defects were not filled. The animals were grouped for sacrifice at 1, 2, 4, and 8 weeks post-surgery. Subsequently, sample blocks were prepared for micro-computed tomography (micro-CT) scanning and histological sectioning. Similar bone formations were observed in all three treatment groups, although the 250–500 μm porcine graft performed slightly better. Rabbit calvarial bone tissue positively responded to porcine grafts and commercial HA/β-TCP, structural analyses showed similar crystallinity and porosity of the porcine and HA/β-TCP grafts, which facilitated bone formation through osteoconduction. These porcine grafts can be considered as graft substitutes, although further development is required for clinical applications.

## 1. Introduction

In past decades, multiple reconstructive procedures have been assessed as periodontal regenerative treatments for deep infrabony defects that are associated with periodontal pockets [1,2] and furcation defects [3] and to prevent bone crest resorption after tooth extraction [4]. Currently, autogenous bone grafts are considered to be the gold standard in bone regeneration procedures because they contain viable osteoblasts, organic and inorganic matrices, and biological modifiers [5]. However, disadvantages of autogenous bone grafts include limited availability, tendency toward partial resorption, requirement of additional surgery, and increased morbidity [5]. Thus, further studies of alternative biomaterials, such as allogenic and xenogenic grafts, and alloplastic materials, are required to determine their efficacy in osteoconduction, osteoinduction, and osteogenesis [3] and to assess their clinical suitability for bone regeneration [6,7,8].

Biphasic calcium phosphate (BCP) is a bioinert and bioactive alloplastic material that comprises mixtures of hydroxyapatite (HA) and beta-tricalcium phosphate (β-TCP) at varying HA/β-TCP ratios [9,10]. Previous studies showed that BCP comprising approximately 60% HA and 40% β-TCP allows control of resorption and maintenance of osteoconductive properties [11,12,13,14,15,16]. Several investigators found that the optimal macroporosity of this material for the ingrowth of bone tissue is between 150 and 500 μm [12]. Accordingly, the commercial biphasic ceramic product, Micro-Macro Biphasic Calcium Phosphate (MBCP, Biomatlante Co. Ltd., Vigneux-de-Bretagne, France), comprises 60% HA and 40% β-TCP and has a complete interconnected porosity of 70% with macropores (300–600 µm) and micropores (<10 µm) at a ratio of 2:1. Among xenogenic materials, osteoconductive and osteoinductive properties of porcine bone substitutes have been reported in numerous histological and morphological studies [17,18,19,20]. However, most of the xenogenic grafts were developed from the bovine bone. The Creutzfeldt–Jakob disease, fetal prion disease in humans, is a problem for the clinical consideration [21]. Therefore, given the similarity of porcine and human genomes, porcine xenograft materials have been widely considered as substitutes for osseous grafts and grafting material within dental implants treatments [18,22,23,24]. In this study, a new porcine graft was developed due to the clinical consideration. The material properties and the bony defect filling effect of the new porcine grafts were investigated in this study.

## 2. Results

### 2.1. Scanning Electron Microscopy

**Figure 1:**
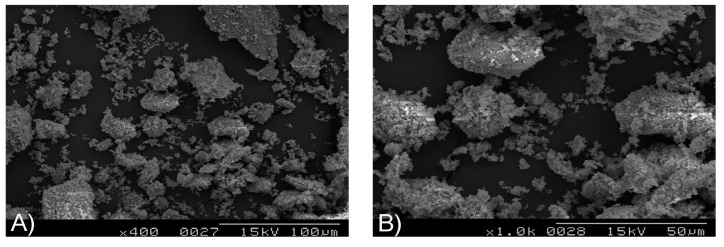
Scanning electron microscope (SEM) images of porcine bone particles: (**A**) Original magnification ×400; (**B**) Original magnification ×1000.

The scanning electron microscopy (SEM) analyses revealed similar structures to those of bovine grafts and HA. Specifically, the porcine grafts had rough surfaces within porous structures of particle surfaces, and induced osteoblastic cell attachment to a 3-dimensional microstructure, and facilitated bone formation. The surfaces also had a spherical morphology comprising a mixture of 250–500 μm and 500–1000 μm particles, which provided a favorable matrix for attachment and proliferation of osteoblasts cells (Figure 1).

### 2.2. Energy Dispersive Spectrometry

The energy dispersive spectrometry (EDS) analyses showed very high accuracy between calcium (Ca) and phosphorus (P), which were present at a Ca/P ratio of 1.668 (28.16% and 16.88%, respectively), and the porcine graft profile resembled that of the HA/β-TCP structure (Table 1).

**Table 1:** Energy dispersive X-ray spectrometer: table of analyzed elements.

Element	Weight (%)	Atomic (%)
C	8.01	13.90
O	44.32	57.77
Na	0.86	0.78
Mg	1.78	1.53
P	16.88	11.37
Ca	28.16	14.65
Total	100.00	100.00

### 2.3. X-ray Diffraction

**Figure 2:**
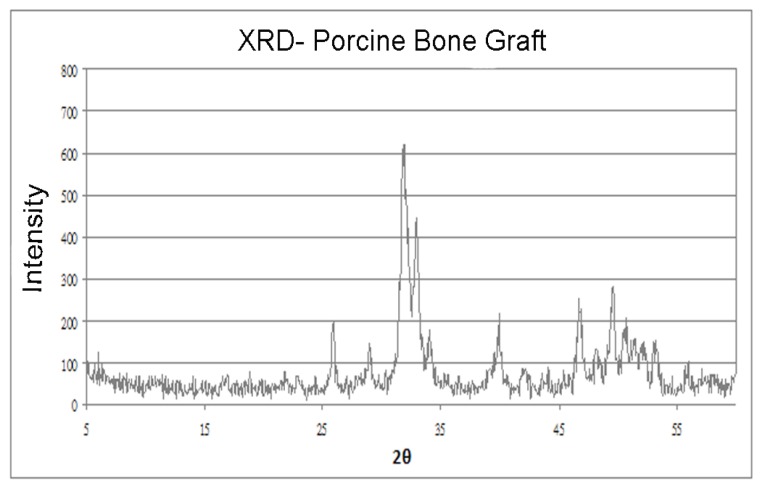
X-ray diffraction: Porcine graft intensities at scattering angles 2θ.

In the XRD analyses, the highest intensity (600 a.u.) of the porcine graft was observed at 27°, and the porcine grafts had peaks of 200 a.u. at 25° and 40°. However, the corresponding wide peak widths indicated low crystallinity (Figure 2).

### 2.4. MTT Proliferation Assay

**Figure 3:**
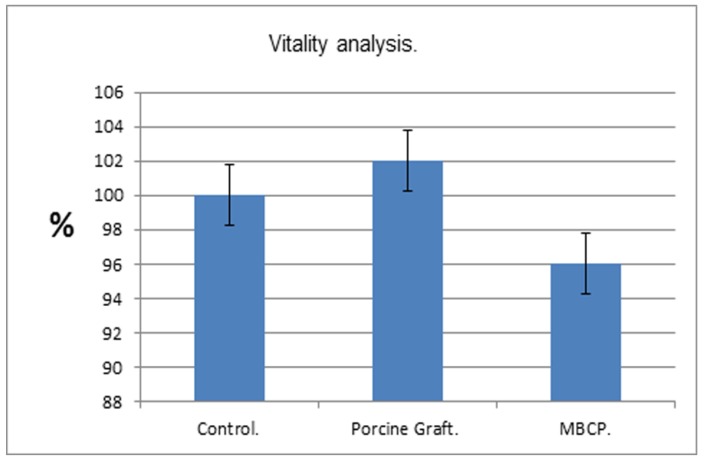
MTT proliferation assays: Lower absorbance values indicate reduced cell proliferation. Porcine grafts enhanced proliferation of osteoblast-like cells.

Absorbance values decreased by 4% in the positive control cells compared with those in the control media. However, osteoblast cell proliferation was 2% greater in the porcine grafts than in the control media with the MG-63 cells progressing from attachment to spreading and showing enhanced cell viability (Figure 3). No significant difference was noted among the different groups.

### 2.5. Micro-CT Scanning

**Figure 4:**
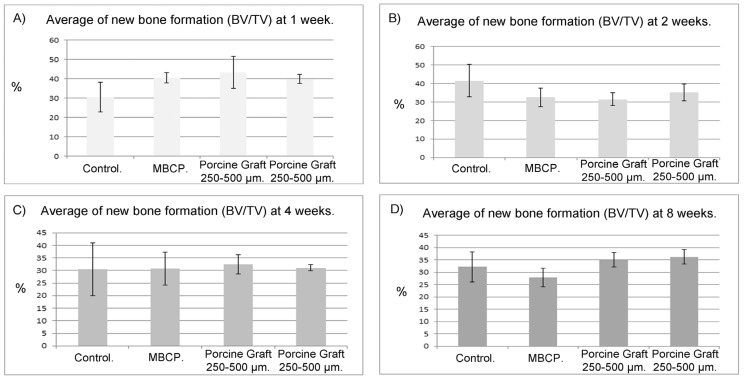
Percentage of bone formation in the total volume of the defect: The bars represent the percentage of bone formation calculated from the relation between bone and tissue volumes in the four calvarial defects at (**A**) one, (**B**) two, (**C**) four and (**D**) eight weeks, in all rabbits.

Porcine graft bone volume densities were calculated using micro-CT and are presented for each time point in Table 2. During week 1, the 250–500 μm porcine graft produced 43% more new bone, whereas MBCP and the 500–1000 μm porcine graft both produced 40% growth and the control media produced 31% growth (*p* < 0.05). New bone growth was reduced in subsequent weeks under all conditions (Figure 4A).

**Table 2:** Micro-computed tomography (micro-CT) assessments of bone formation. Data are presented as means and standard deviations.

Micro CT Average-SD Values for Newly Formed Bone				
Time	Control	MBCP	Porcine Graft 250–500 μm	Porcine Graft 500–1000 μm
Week 1	30.57 ± 7.57	40.42 ± 2.64	43.29 ± 8.37	40 ± 2.33
Week 2	41.57 ± 8.71	32.59 ± 5.05	31.52 ± 3.51	35.24 ± 2.33
Week 4	30.38 ± 10.54	30.7 ± 6.5	32.47 ± 3.83	31.05 ± 1.3
Week 8	31.55 ± 7.25	27.81 ± 3.86	35.02 ± 2.92	36.19 ± 2.85

The rate of new bone production was the greatest in the control group during week 2, with a 42% increase compared with 33%, 32%, and 35% increases in the MBCP, 250–500 μm porcine graft, and 500–1000 μm porcine graft groups, respectively. New bone formation in all filled defects was less in week 2 than in week 1 (Figure 4B).

During week 4, the 250–500 μm porcine graft produced 33% new bone, which did not significantly differ with that of the MBCP (31%) or 500–1000 μm porcine graft (31%) groups. In contrast, new bone growth was only 12% in the control group (Figure 4C).

After eight weeks, the 250–500 μm porcine graft produced 35% new bone growth similar to that seen at weeks 1 and 4 and was superior to the other treatments. MBCP only produced 28% new bone growth, which was equal to that in the control group. The 500–1000 μm porcine graft showed improved performance from week 4, with 36% new bone formation at week 8 (Figure 4D).

### 2.6. Histological Examination

**Figure 5:**
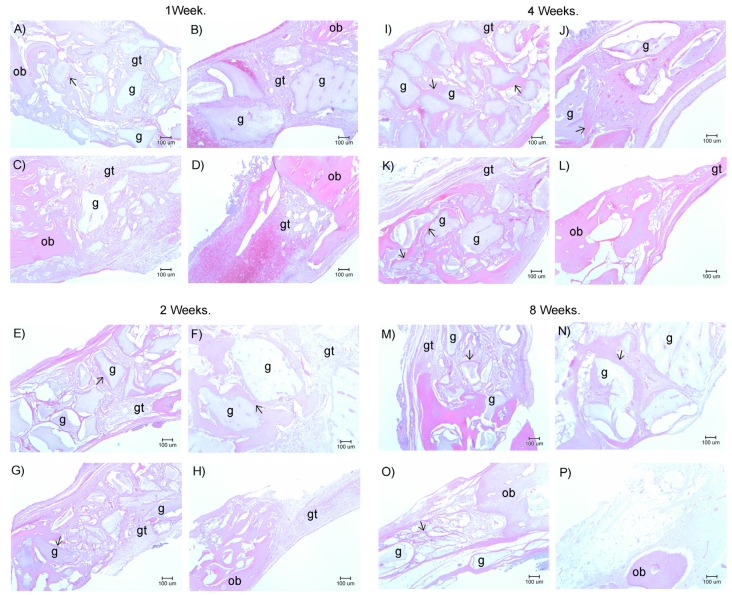
Histology of porcine graft in rabbit calvarial bone defect. Panoramic ×40 Hematoxylin–Eosin stain images of the different defects at one, two, four, eight weeks. (**D**, **H**, **L**, **P**) Control defects; (**A**, **E**, **I**, **M**) MBCP; (**C**, **G**, **K**, **O**) Porcine Graft 250–500 μm; (**B**, **F**, **J**, **N**) Porcine Graft 500–1000 μm. Arrows indicate new bone formation, g = graft, gt = granulation tissue, ob = old bone.

Photomicrographs of H&E stained sections from the rabbit calvaria showed granulation tissue in the control defect and in the defects filled with MBCP, 250–500 μm, and 500–1000 μm porcine grafts. Specifically, the defects contained particles in direct contact with woven bone at the border and granulation tissue in the middle of the defect, and all filled defects presented acceptable bone formation (Figure 5A–D).

Light miscroscope photographs of the H&E stained sections from the week 2 showed similar patterns to those from the week 1. However, the granulation tissue was replaced with loose fibrous connective tissue, and although this connective tissue encapsulated some graft particles, other particles were surrounded by new bone (Figure 5E–H).

At the week 4, the analyses showed newly formed bone in contact with biomaterials, and whereas new bone was observed in the contours of the control defects, more new bone was observed around the particles in the MBCP-filled defects and around and across particles in defects filled with 250–500 μm and 500–1000 μm porcine grafts. All the defects had less connective tissue at this time point than at previous time points. (Figure 5I–L).

At the week 8, the control defects showed partial closures with bone around the defect and well organized connective tissue in the middle. Moreover, the bone areas increased with decreasing MBCP, 250–500 μm, and 500–1000 μm porcine graft areas. Finally, the bone was more mature at week 8, and borders between newly formed bone and graft materials were easily observed (Figure 5M–P).

## 3. Discussion

In comparison with models using self-contained and/or dehiscence-type defects, the critical-size defect model represents a challenging situation for bone formation because bone proliferation can only occur from the borders of the calvarial defect [25]. Hence, this model has been used to study regeneration of defects with and without the use of barrier membranes. In the present study, bone formation was assessed in the rabbit calvarial critical-size defects without barrier membranes in the presence and absence of novel porcine graft materials with varying particle sizes.

Implantation of 250–500 μm and 500–1000 μm porcine grafts into the rabbit calvarial bone led to equal or better healing than implantation of MBCP. Moreover, micro-CT scanning demonstrated greater percentage increases in bone volume density in the presence of the three graft materials than in the control defects. Micro-CT scanning provides accurate determinations of both cancellous and cortical bone and of spatial distributions of scaffold mineralization, particularly in calvarial defects [26]. Nonetheless, histological examinations are considered the gold standard for assessments of healing after fracture wounds and surgical procedures [27]. Thus, bone formation were investigated using micro-CT scanning and light microscopy. In the microscopic observation, the new bone formations were found around the surface of the porcine graft, as Albrektsson *et al*. (2001) indicated osteoconduction means that bone grows on a surface [28]. The histological results demonstrate the osteoconductive potential of the porcine graft. The similar results also showed as qualitative description in the micro-CT observation and further validated the application of micro-CT scanning for measurements of new bone formation.

In agreement with the present data, Calvo *et al*. (2013) showed biocompability, bioabsorbability, and osteoconductive characteristics of porcine grafts that did not inhibit normal bone repair [18]. Moreover, previous studies showed promising clinical application of porcine bone [5,6,29], although sinus augmentation from subsequently placed dental implants revealed condensation properties in one-year histological analyses, indicating absorption of the material with time [19]. In agreement, Barone *et al*. (2012) performed six-month hystomorphometric evaluations of maxillary sinus augmentation following implantation of prehydrated corticocancellous porcine bone, and showed excellent osteoconductive properties that facilitated sinus augmentation. Moreover, porcine bone showed a high percentage of reabsorption after six months, potentially reflecting the collagen contents and porosity of the graft material [30].

In the present X-Ray Diffraction (XRD) analyses, the porcine grafts exhibited similar Ca:P ratios to those of human bone and showed a maximum intensity of 600 a.u. at 27°, similar to that in HA (21°). Moreover, peaks of 200 a.u. were observed at 25° and 40°, which correspond with those in adult human and bones from 26 day old fetuses, although peak widths indicated low crystallinity at these angles. Nonetheless, these similarities of porcine graft materials and human bones indicate that these graft materials facilitate the production of new bone (Figure 2).

Slotte *et al*. (2013) demonstrated considerable gain of new bone in clinical cases of maxillary sinus bone augmentation using porcine bone and showed reabsorption and replacement with new bone over time [31]. In the present study, formation of new bone was observed *in vivo* in bone defects that were filled with 250–500 μm and 500–1000 μm porcine graft materials, which were slowly reabsorbed. Similar efficacies of these materials may reflect interporotic connections, synthetic temperatures (800 °C), and particle sizes of 100 μm, which is regarded as the minimum pore size for guaranteed bone growth. Go *et al*. (2013) [32] investigated the efficacy of low crystallinity materials with high surfaces areas, large pore sizes, and large pore volumes, which are crucial properties for osteoblast-like cell affinity and for degradation of graft materials during bone regeneration. In that study, differences in chemical and physical properties of porcine-derived xenografts that were heat treated at 400 °C and 1200 °C were described. Heat treatment at 400 °C led to slightly greater increases in new bone volume (45% *vs*. 43%), and the resulting porous structures had larger surface areas and pore volumes than those following heat treatment at 1200 °C [32]. Regardless of particle sizes, the present xenogenic porcine grafts were made at 800 °C and demonstrated identical crystallinity on XRD analyses. However, concomitantly wider peaks were indicative of limited crystallinity of these grafts, which may facilitate direct decomposition by osteoclasts and remodeling of endogenous bone materials [32]. Although crystallinity did not differ between the porcine grafts of differing particle sizes, bone formation was slightly greater with the 250–500 μm porcine graft material. However, both 250–500 μm and 500–1000 μm graft materials were synthesized at temperatures that produce mesopores and macropores and are, therefore, both viable substitutes for autogenous grafts in bone regenerating treatments.

The use of porcine xenogenic grafts in humans carries the risk retroviral transmission from pigs to humans. However, numerous studies showed no infrequent pig endogenous retrovirus transmissions, indicating the safety of transplantation into humans. Nonetheless, these risks require examination in animals models of pig to human xenotransplantations and in clinical studies [17]. Kim *et al*. (2013) assessed the safety of bovine-derived graft biomaterials that may transmit bovine spongiform encephalopathy prions. However, this risk remains poorly characterized, and although bovine derived bone materials are effective for the treatment of human intrabony periodontal lesions, these materials are dangerous in humans [21]. Taken together, the present data indicate the efficacy and safety of porcine bone grafts and warrant further investigation in human studies.

## 4. Materials and methods

### 4.1. Biomaterials

The biphasic ceramic material MBCP comprises 60% HA and 40% β-TCP and has a complete interconnected porosity of 70%, comprising macropores of >10 µm and micropores of <10 µm at a 2:1 ratio [33].

The present porcine grafts were produced with particle sizes of 250–500 μm and 500–1000 μm at Taipei Medical University. Porcine graft material with a high porosity was produced using cortical porcine bone. In this procedure, pig bones were collocated in water and were settled at 300–400 °C for 15–30 min to remove fat, residual meat, and other soft tissues. Subsequently, the bones were re-cut into 5–10 mm pieces and were immersed in 0.1–0.5 M hydrochloric acid (HCl) for 5–10 min to etch and remove proteins. The bones were then rinsed with deionized water and dried and were subjected to the following temperature gradients in crucibles with a heating process:

- 5 °C/min heated to 100 °C and then maintained for 30 min;
- 5 °C/min heated to 300 °C and then maintained for 60 min;
- 10 °C/minute heated to 800 °C and then maintained for 120 min.

The samples were then allowed to cool to room temperature, and the resulting products were sieved to the desired particle size in a mortar. Finally, bone aliquots were placed into glass bottles and were sterilized using γ-rays.

### 4.2. Porcine Graft Analysis

#### 4.2.1. Scanning Electron Microscopy and Energy Dispersive Spectrometry

Elemental analyses of porcine graft samples were performed using a scanning electron microscope (SEM) equipped with an energy dispersive X-ray spectrometer (EDS).

#### 4.2.2. X-ray Diffraction

Porcine graft diffraction patterns were analyzed using X-ray diffraction (XRD), and crystal structures and constituent materials were determined.

#### 4.2.3. Cell Proliferation (MTT Assay)

Cell metabolic activity was evaluated according to succinic dehydrogenase (SDH) activity using the spectrophotometric methyl tetrazolium assay (MTT assay) without washing steps. The main advantages of the colorimetric assay are rapid, precision, and without any radioisotope [34]. In these experiments, Dulbecco’s Modified Eagle’s Medium (DMEM-S) solutions containing Phosphate Buffered Saline (BLS) + polysialyltransferases (PST) with or without (control) porcine or commercial graft materials were incubated in a humidified incubator at 37 °C. After 72 h, the media solutions were separated from porcine and commercial graft materials and were placed on MG-63 cells, which were then allowed to recover for 24 h. Cell metabolic activity was then analyzed in 12 cell cultures for each media solution.

The MG-63 cells were seeded (30,000 cells/cm^2^) onto 24-well plates (Costar Corporation, Cambridge, MA, USA) and were maintained in a humidified incubator with 5% CO_2_ and 95% air at 37 °C for 72 h. Thereafter, the culture medium was aspirated, and all media solutions were added to the cell culture wells. After exposure for 24 h, the media solutions were aspirated and replaced with 500 µL of culture medium (α-MEM) and 500 µL of MTT solution (5 mg/mL phosphate buffered saline-PBS) and were incubated at 37 °C for 24 h. Thereafter, the solution was replaced with 500 µL of acidified 0.5% Triton X-100, and absorbance was measured at 570 nm using a spectrophotometer (Plate Chameleon Multilabel Detection Platform; HIDEX Co. Ltd., Turku, Finland).

Absorbance values were standardized to solutions of tetrazolium bromide, and toxicity was determined according to cell survival. The data were analysis with a Student *t*-test.

#### 4.2.4. Statistical Analysis

The non parametric Kruskal–Wallis test was used to analyze the histomorphometric from the Micro CT data among experimental times within each tested material. MTT assay were analyzed by Student *t*-test. The significant level was set at *p* < 0.05.

## 5. Surgical Procedure

**Figure 6:**
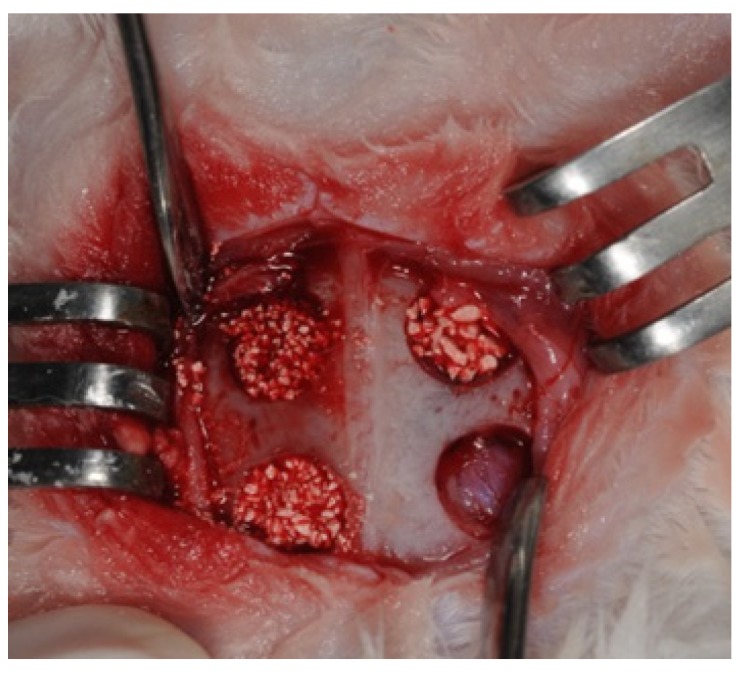
Graft materials and calvarial critical-size defects. The four calvarial critical-size defects were filled with 250–500 μm porcine graft (upper left), 500–1000 μm porcine graft (upper right), or MBCP (lower left), and the lower right defect was used as an unfilled control.

Surgical procedures were performed in 20 adult male New Zealand white rabbits with a mean age of 10 weeks and a mean weight of 2.1 kg. The animals were housed in cages at 19 °C and 55% humidity in the Taipei Medical University Laboratory Animal Center and were fed standard rabbit chow and water *ad libitum*. Anesthesia was administered using an intramuscular injection of Zoletil 50 (50 mg/mL) at a dosage of 15 mg/kg into the gluteal region, and surgery was performed in the sedated animals after 10 min. The calvarial region was then shaved, draped, and sterilized using iodine, and 1.8 mL of 2% lidocaine with epinephrine 1/100,000 was then injected as a hemostatic. Subsequently, 2 cm longitudinal vertical skin midline incisions were made, periosteums were retracted to expose calvarial bones, and four critical calvarial defects of 5 mm diameter and 3 mm depth were prepared using a trephine bur (3I implant innovation, Palm Beach Gardens, FL, USA) bilaterally in the parietal and frontal bones of each rabbit. The upper left, upper right, and lower left defects were filled with 250–500 μm porcine graft, 500–1000 μm porcine graft, and HA/β-TCP (MBCP; Biomatlante biologics solutions), respectively, and the lower right defects were used as unfilled controls. The animals were kept in cages under surveillance for the first 24 h and were then examined every 3 days for 2 weeks, and weekly thereafter [25,35] (Figure 6).

The animals were grouped for sacrifice at 1, 2, 4, and 8 weeks after surgery. Euthanasia was performed by CO_2_ asphyxiation 10 min after the intramuscular injection of Zoletil 50 (50 mg/mL) at 15 mg/kg into the gluteal region. Subsequently, a section was made through the middle of the frontal plane between the 4 filled critical defects. Samples blocks were prepared in formalin and micro-computed tomography (micro-CT) scanning analyses were performed within 2 weeks using Skyscan 1076 (Skyscan, Antwerp, Belgium).

After setting the micro-CT images, coronal images of the center and peripheral areas of the defect were saved in the database and 2-dimensional (2D) morphological analyses were performed for 64 samples. Among these, 8 were not analyzed because of premature deaths of two animals.

**Figure 7:**
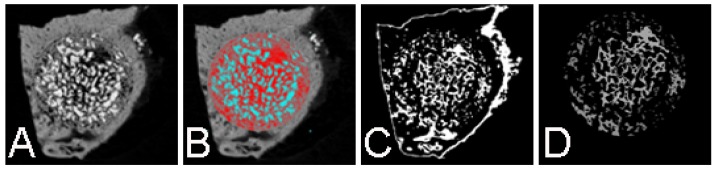
Morphometric analyses in grayscale density using Skyscan 1076 Skyscan CTan software. Morphometric analyses were done in grayscale density using Skyscan 1076 Skyscan CTan software (**A**) Raw image, coronal view; (**B**) Determine region of interest; (**C**) Binari selection of the sample; (**D**) Morphometric analysis.

Morphometric parameters were calculated on individual binarized cross-section images, and 2D morphometric parameters were determined slice-by-slice and were then integrated over multiple slices. After integration of whole volumes of interest (VOI), percentages of VOI occupied by binarized solid objects were calculated. This parameter comprises percentages of the total volume (TV) of VOI and the total binarized volume (BV) of objects within VOI. However, this relationship is only relevant if the studied volume is fully contained within a well-defined biphasic region of solid and space, such as the trabecular bone region, and does not extend into or beyond the boundary of the object, such as the cortical boundary of the bone sample. Moreover, measured percent volumes are dependent on the criteria for selection of VOI. Thus, binary selections of samples from the morphometric analyses were made according to grayscale density between units of 20 and 80. The morphometric analyses were performed using Skyscan 1076 Skyscan CTan software according to the manufacturer’s instructions (Figure 7).

Tissue sections were taken from all micro-CT sample blocks using the following procedures:

- Decalcification: The bone specimens were trimmed to a thickness of less than 0.5 cm (to accelerate decalcification) and were decalcified in fresh fluid. Decalcification times, specimen thicknesses, temperature, decalcification solution freshness, and block samples’ decalcification conditions were recorded.
- Washing with water: After decalcification, the specimens were washed in running water for several hours to neutralize the strongly acidic decalcifying solution.
- Dehydration and embedding: The bone tissues were dehydrated in alcohol and were then embedded in liquid paraffin.
- Sectioning: Slice thicknesses were set at 5–7 μm.
- Staining: Hematoxylin and Eosin (H&E) staining was performed on all paraffin embedded tissues.
- Visualization: The histological slides were viewed using an optical microscope (Olympus BH-2, Tokyo, Japan), and the images were captured at 40× magnification using a camera SPOT idea tm Camera that was connected to the microscope and were analyzed using the corresponding software (SPOT imaging software, Sterling Heights, MI, USA).

## 6. Conclusions

The present porcine grafts showed great promise and resembled human bone in the micro-CT, XRD, and histological analyses, and promoted cell survival and growth of new bone. The rabbit calvarial bone tissue responded well to both the 250–500 μm and 500–1000 μm porcine grafts, which had similar efficacy to that of commercial HA/β-TCP (MBCP). The present observations also indicate that porcine grafts do not interfere with wound healing and promote bone formation through osteoconduction, warranting further investigation as graft substitutes. Although minimal graft reabsorption was observed in this study, longer experiments may show sufficient resorption in humans.
